# The Major Roles of DNA Polymerases Epsilon and Delta at the Eukaryotic Replication Fork Are Evolutionarily Conserved

**DOI:** 10.1371/journal.pgen.1002407

**Published:** 2011-12-01

**Authors:** Izumi Miyabe, Thomas A. Kunkel, Antony M. Carr

**Affiliations:** 1 Genome Damage and Stability Centre, University of Sussex, Brighton, United Kingdom; 2 Laboratory of Molecular Genetics and Laboratory of Structural Biology, National Institute of Environmental Health Science, National Institutes of Health, Research Triangle Park, North Carolina, United States of America; The University of North Carolina at Chapel Hill, United States of America

## Abstract

Coordinated replication of eukaryotic genomes is intrinsically asymmetric, with continuous leading strand synthesis preceding discontinuous lagging strand synthesis. Here we provide two types of evidence indicating that, in fission yeast, these two biosynthetic tasks are performed by two different replicases. First, in *Schizosaccharomyces pombe* strains encoding a *polδ-L591M* mutator allele, base substitutions in reporter genes placed in opposite orientations relative to a well-characterized replication origin are strand-specific and distributed in patterns implying that Polδ is primarily involved in lagging strand replication. Second, in strains encoding a *polε-M630F* allele and lacking the ability to repair rNMPs in DNA due to a defect in RNase H2, rNMPs are selectively observed in nascent leading strand DNA. The latter observation demonstrates that abundant rNMP incorporation during replication can be tolerated and that they are normally removed in an RNase H2-dependent manner. This provides strong physical evidence that Polε is the primary leading strand replicase. Collectively, these data and earlier results in budding yeast indicate that the major roles of Polδ and Polε at the eukaryotic replication fork are evolutionarily conserved.

## Author Summary

It is important to understand the architecture of the DNA replication machinery and whether this is common to all organisms. Recent work in *Saccharomyces cerevisiae* has genetically assigned specific DNA polymerases to leading and lagging strand DNA synthesis, Polε and Polε respectively. In this manuscript, we use a similar genetic assay to demonstrate that, in the highly evolutionarily diverged yeast *Schizosaccharomyces pombe*, Polδ is similarly responsible for lagging strand synthesis. Importantly, we establish a novel physical assay, the incorporation of rNMPs into newly replicated DNA, which demonstrates that Polε is responsible for leading strand synthesis and does not contribute significantly to lagging strand replication. These data strongly support and consolidate the interpretation of previous genetic data and suggest that the division of labour between polymerases is conserved through evolution.

## Introduction

Three DNA polymerases, Polα, Polδ, and Polε, are required for efficient genome replication in eukaryotes [1], [2]. The Polα holoenzyme complex has both primase activity and DNA polymerase activity and is required to initiate each DNA synthesis reaction. The primase subunit first synthesizes a short RNA primer of ∼10 nucleotides and the DNA polymerase subunit then extends this primer using dNTPs for a further 20–30 nucleotides, thus initiating DNA replication. Polδ or Polε then substitutes for Polα and perform the bulk of DNA replication by elongating these primers.

Genomic DNA is replicated faithfully during every cell cycle with an error rate of approximately 1 in 10^−10^ errors per base pair, ensuring that the genetic blueprint is transmitted largely unaltered through the generations. In eukaryotic cells, DNA replication is initiated bi-directionally from many replication origins. Because of the antiparallel structure of DNA, one strand (leading strand) is replicated continuously in the same direction of the replication fork, while the second strand (lagging strand) is synthesized discontinuously in the opposite direction to that of replication fork progression. The relatively small (200–1000 base) stretches of DNA synthesized during lagging strand replication are known as Okazaki fragments and are rapidly processed and ligated to complete lagging strand replication. The fidelity of replication is ensured by the nucleotide selectivity of replicases to achieve error rates of 10^−4^–10^−5^, by exonucleolytic proofreading during replication to increase fidelity about 100-fold, and by post-replication DNA mismatch repair to further increase fidelity and lower the mutation rate to 10^−8^–10^−10^ [3].

Polα, Polδ, and Polε all belong to the B family of DNA polymerases. The structure of the active site of B family DNA polymerases is highly conserved throughout evolution. As for most polymerases, the precise geometry of the polymerase active site ensures that mismatches are largely precluded from incorporation [4]. The importance of polymerase active site geometry to replication fidelity is illustrated by the fact that substitutions of conserved active site residues often reduce DNA synthesis fidelity. Relevant to the present study are substitutions in *Saccharomyces cerevisiae* Polε and Polδ (M644G and L612M, respectively) that increase error rates during DNA synthesis *in vitro* and also result in elevated spontaneous mutation rates *in vivo* [5]–[8]. These polymerases have particular value for studies of replication fidelity *in vivo* because their error rates are preferentially elevated for only one of two possible mismatches that could result in a particular base substitution in a cell. For example Polδ L612M preferentially generates T-dGTP rather than A-dCTP errors, and this preference yields strand specific A–T to G–C mutations during duplex DNA replication *in vivo*. These biased error rates result in asymmetric mutation profiles in a *URA3* reporter gene that is replicated in only one direction due to its close proximity to an active origin. When present in each of the two possible *URA3* orientations relative to the origin, the mutational patterns observed in strains harboring the *pol2-M644G* (polε) and *pol3-L612M* (polδ) mutator alleles imply that *S. cerevisiae* Polε and Polδ are the primary leading strand and lagging strand replicase, respectively [9], [10].

The goal of the present study is to identify the major leading and lagging replicases in the fission yeast *Schizosaccharomyces pombe*. To investigate Polδ, we took advantage of the fact that both *S. cerevisiae* Polδ L612M [6] and its human equivalent, Polδ L606M [11], [12] have been shown to have biased DNA synthesis fidelity. Here we report that *Schizo. pombe* Polδ L591M generates asymmetric mutation profiles *in vivo* that are consistent with Polδ being the primary lagging strand replicase in *Schizo. pombe*. To investigate Polε, we attempted to generate a *Schizo. pombe* Polε mutation (*polε-M630G*) equivalent to that previously studied in *S. cerevisiae* (encoding Polε M644G). *Schizo. pombe* strains with this substitution were not viable. We therefore generated a different allele, *polε-M630F*, because substitution of phenylalanine at the equivalent active site residues in *S. cerevisiae* Polα [13] and Polζ [14] are viable and have elevated spontaneous mutation rates. We show here that the *Schizo. pombe polε-M630F* allele is also viable and a spontaneous mutator. Although it did not display a suitable asymmetric mutation profile for strand assignment, we were able to exploit a second infidelity parameter for strand assignment, the propensity to incorporate rNMP into DNA. Previous studies have demonstrated that during DNA synthesis *in vitro* and *in vivo*, *S. cerevisiae* Polε M644G incorporates greater amounts of rNTPs into DNA than does wild type Polε [15], [16]. Here we exploit this same promiscuity with the *Schizo. pombe polε-M630F* mutant, to provide a physical demonstration that the majority of leading strand synthesis in *Schizo. pombe* is performed by Polε.

## Results

### Approach

Which DNA polymerase replicates which strand has only been determined in the budding yeast *S. cerevisiae* [9], [10]. We thus wished to determine if this division of labour between the main replicative polymerases is conserved in the distantly related eukaryote, the fission yeast *Schizo. pombe*. Our strategy was to establish the direction of replication for a specific locus, to create mutants in the genes encoding two replicative polymerases, Polδ and Polε, that exhibit specific and characteristic profiles of misincorporation, and to use these to assign each polymerase to one or the other strand (or both) based on the profile of misincorporation at the directionally replicated loci.

The catalytic subunits of Polδ or Polε are encoded by the *cdc6* (*pol3*) and the *cdc20* (*pol2*) genes, respectively. For clarity, here we simply refer to them as *polδ* and *polε*. We employed recombination-mediated cassette exchange (RMCE) to create strains that harbor each specific mutant polymerase [17]. Mutant genes introduced into the genome by this method are flanked by lox (P and M3) sequences. Thus, we also created control strains (*pol^+^*) that have the gene encoding the wild-type polymerase flanked by the same lox sites.

### Direction of DNA Replication at the *ura4* Locus

**Figure 1:**
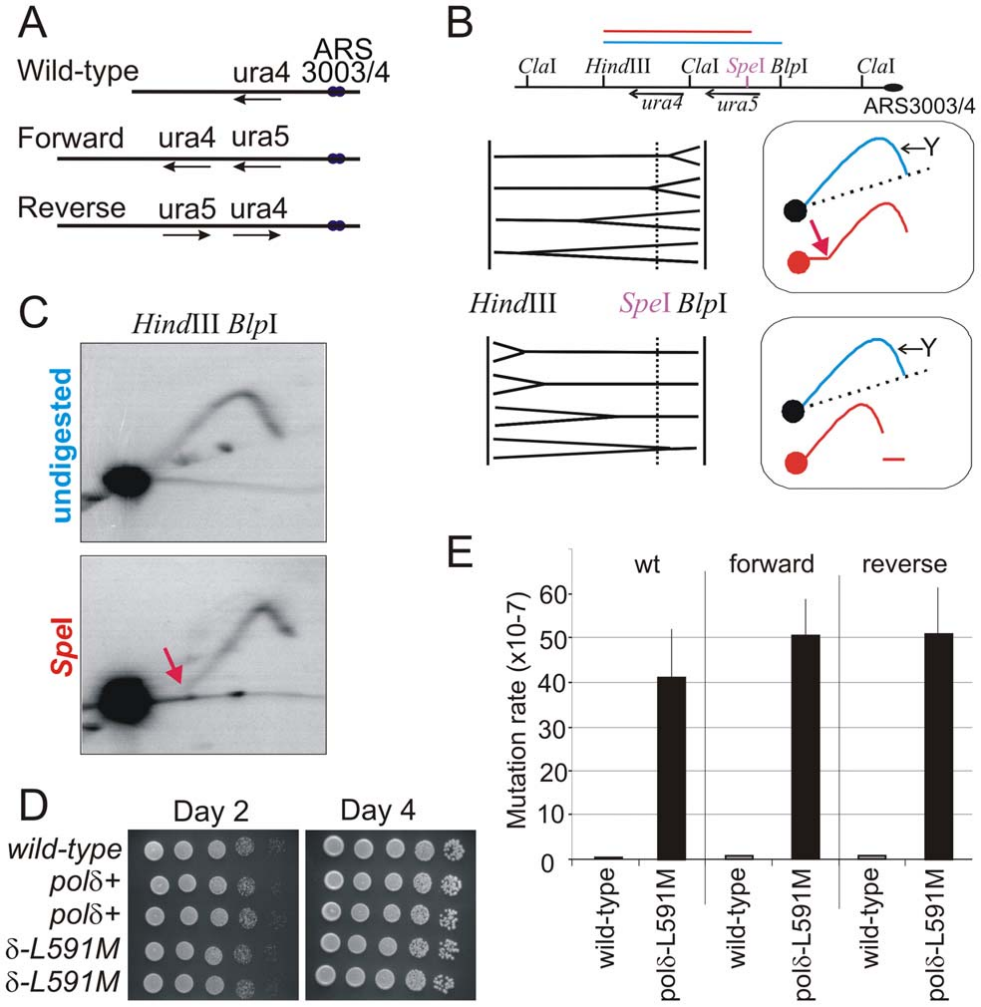
Polδ L591M shows mutagenic strand bias. A. Schematic of the wild type *ura4^+^* locus and the two versions of the modified *ura4^+^:ura5^+^* loci. Forward: the transcribed strand of both *ura4* and *ura5* corresponds to the lagging strand when replicated from *ars3003/3004*. Reverse: the orientation of the *ura4:ura5* sequences are switched so that the transcribed strand corresponds to the leading strand when replicated from *ars3003/3004*. Loss of either *ura4* or *ura5* function results in 5-FOA resistance. B. The direction of replication at the modified *ura4* ^+^ locus. Top: Schematic of the *ura4* ^+^:*ura5* ^+^ locus. Bottom: the principle of directional 2D-gel analysis. Asymmetric digestion of the *Hind*III-*Blp*I fragment with *Spe*I between the running of the first and second dimensions will result in a shift of the Y-arc. The position of the Y arc is indicated by an arrow. The direction of shift is dependent on the direction of replication. C. Comparison of replication intermediates within the *Hind*III-*Blp*I region either without (undigested) or after *Spe*I digestion between dimensions (*Spe*I). The shifted Y arc following *Spe*I digestion is indicated with a red arrow, the equivalent of which is also shown in the top frame of panel B. The majority of replication forks run from right to left, corresponding with the efficient initiation from *ars3003/ars3004* on the right. D. Cell growth of wild type, *polδ^+^* (wild-type *polδ* flanked by lox sites) and *polδ-L591M*. Serial dilutions of cells were spotted on YEA plates, incubated (30°C) for 2 or 4 days and photographed. E. Spontaneous mutation rates in each of the indicated backgrounds for *polδ^+^* and *polδ-L591M*. Error bars show standard deviation.

The *Schizo. pombe ura4^+^* gene allows for both positive and negative selection. Selecting for loss of *ura4* function is achieved by growth on medium containing 5-fluoroorotic acid (5-FOA), which identifies loss-of-function mutants. However, mutations in either the *ura4* or the *ura5* genes of *Schizo. pombe* confer 5-FOA resistance, and it has been reported that greater than 50% of spontaneously arising 5-FOA resistant clones harbor mutations in *ura5* [18]. In wild type cells, *ura4^+^* is located on chromosome III while *ura5^+^* is located on chromosome II. Therefore, to efficiently identify mutations at a single chromosomal location that confer 5-FOA resistance, we created two artificial loci where *ura5^+^* was placed adjacent to *ura4^+^* on chromosome III. These differ only in the orientation of the *ura4^+^*:*ura5^+^* fragment (Figure 1A). We confirmed that this novel *ura5^+^*:*ura4^+^* fragment does not function as a replication origin by demonstrating it would not support maintenance of plasmid sequence in cells. We also deleted the genomic *ura5^+^* gene on chromosome II, so that resultant *ura4^+^:ura5^+^ Δura5* strains have only one copy of the *ura4^+^* and *ura5^+^* genes.

The *ura4* ^+^ *:ura5^+^* locus is on Chromosome III, near two autonomous replicating sequences; *ars3003/3004*. Both the *ars3003* and *ars3004* sequences have been well characterized and are known to be highly efficient at initiating replication [19], [20]. However, more than 50% of *Schizo. pombe* intergenic regions have the potential to function as origins of replication [21]. Thus, to experimentally determine the direction of DNA replication at the *ura4^+^:ura5^+^* locus, we employed the method of directional 2-D gel electrophoresis [22]. DNA from an asynchronous population of cells is first digested with *Hind*III and *Blp*I and fragments separated in the first dimension without ethidium bromide. The lane is then excised and digested with *Spe*I, which cleaves within the *Hind*III-*Blp*I fragment containing the *ura4^+^* and *ura5^+^* genes. This DNA is then subjected to the second dimension of electrophoresis in the presence of ethidium bromide and DNA in the gel is transferred to a membrane for Southern blot analysis with the *ura4-containing Hind*III-*Spe*I fragment. The results revealed the direction of DNA replication, as illustrated in Figure 1B. Most of detectable replication intermediates show the pattern consistent with DNA replication moving from right to left (Figure 1C, see red arrow bottom panel: its equivalent is similarly indicated in the top panel of Figure 1B). Thus, we conclude that a leftward replication fork replicates the *ura4^+^:ura5^+^* locus in the majority of cells.

### Characterization of a *polδ-L591M* Mutant

We then created the *polδ-L591M* mutant using RMCE. *Schizo. pombe* Polδ L591 is equivalent to *S. cerevisiae* Polδ L612. *polδ-L591M* cells grow as well as wild type cells (Figure 1D), demonstrating that this mutant of Polδ is proficient for DNA replication *in vivo*. In wild type and *ura4^+^:ura5^+^ Δura5* backgrounds, *polδ-L591M* showed a strong mutator phenotype (Figure 1E). Spontaneous mutation rates are elevated ∼100 fold in *polδ-L591M* (4–5×10^−6^/cell division) compared with that in *polδ^+^* (4–7×10^−8^).

### Mutational Bias in *polδ L591M* Strains

The elevated mutation rates indicate that most of the mutations seen in *polδ -L591M* cells reflect the error specificity of this mutant polymerase, rather than background mutations. As shown in Table 1, more than half of mutations were point mutations, consistent with elevated base misincorporation observed *in vivo* for the equivalent *S. cerevisiae* strain and *in vitro* for the corresponding mutant version (L612M) of *S. cerevisiae* Polδ [5], [6], [23] and human (L606M) Polδ [11], [12]. In addition to point mutations, we observed a variety of duplication and deletion mutations. All of these deletions and duplications were observed at repetitive DNA sequences. More than half of the deletions were >100 bp, while the majority of duplications were <100 bp (Table S1). Possible mechanisms by which such mutations may arise are addressed in the Discussion.

**Table 1:** 5-FOA resistant mutants from *polδ-L591M.*

	polδ-L591M		polδ+	
	forward	reverse	forward	reverse
Base substitutions	71	97	83	101
1 base deletions	51	37	8	5
1 base insertions	8	7	10	4
Duplications	47	45	6	6
Deletions	18	13	13	12
Others	41	30	3	3
**Total**	**236**	**229**	**123**	**131**

**Figure 2:**
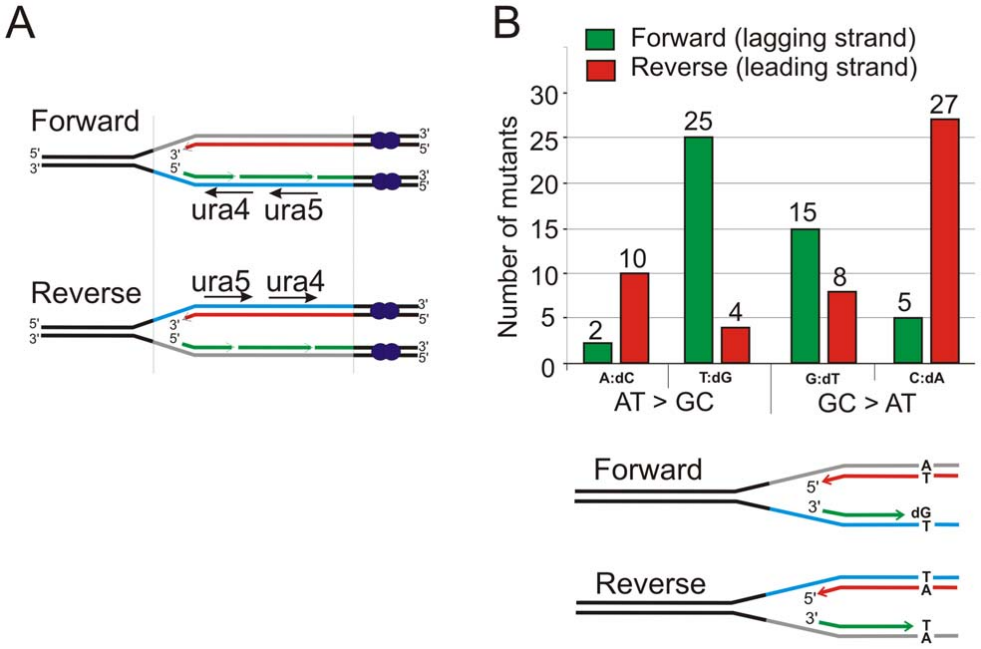
Strand bias for *polδ-L591M*. A. Schematic of replication through the Forward and Reverse *ura4^+^*:*ura5^+^* loci. Leading strand synthesis shown in red, lagging strand synthesis is shown in green. The transcribed strand is shown in blue for reference. B. Top: the relative numbers of AT>GC and GC>AT mutations identified, classified as resulting from either A:dC or T:dG mispairing (AT>GC) and G:dT or C:dA mispairing (GC>AT). Bottom. Schematic illustration of replication of a specific A:T base pair in both orientations. If the lagging strand polymerase, but not the leading strand polymerase, is prone to mispairing dG opposite T during incorporation, but not dC opposite A, then this A:T base pair will mutate to G:C more frequently in the forward orientation than the reverse orientation.

Summary of all 5-FOA resistant mutations seen in the *pol*δ*-L591M* mutant and the *polδ* ^+^ strains in the Forward and Reverse *ura4^+^:ura5^+^* backgrounds. Single base changes for *polδ-L591M* are indicated above or below the corresponding sequence in Figure 2.

**Figure 3:**
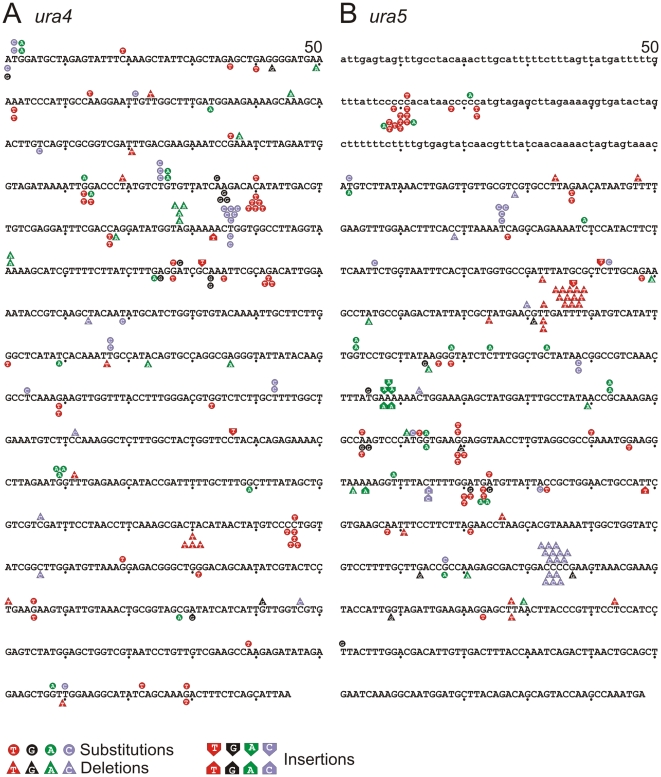
Mutation spectra. A. *ura4* and B. *ura5*. The promoter region of *ura5* (lower case) and the ORF's of both *rad4* and *ura5* (upper case) are shown. The position of each mapped mutation is indicated. Mutations arising in the “forward” background (transcribed strand replicated by lagging strand synthesis) are shown above the sequence, whereas the mutations arising in the “reverse” background (transcribed strand replicated by leading strand synthesis) are shown below the sequence.

Among the point mutations, transition mutations showed significant strand dependence for misincorporation. Figure 2 and Table 2 show the predicted mispairs formed during synthesis of the transcribed strand, which corresponds to lagging or leading strand synthesis in the Forward or Reverse strains, respectively (illustrated in Figure 2A). A:T to G:C mutations can result from either A:dCTP mismatches or T:dGTP mismatches. Depending on which template strand is copied by the mutated polymerase, this will give a bias of mutation resulting in a spectra dependent on the orientation of the DNA sequence (see Figure 2B). We observed that, for A:T to G:C changes, T:dG mispairing is 12.5 fold more frequent than A:dC mispairing in the Forward strain, while A:dC is more frequent in the Reverse strain. Since the misincorporation rate of the corresponding mutant *S. cerevisiae* and human polymerases are much higher for T:dG than for A:dC [6], [12], the results in Figure 2 imply that Polδ preferentially replicates the lagging strand template. A similar bias was also observed for G:C to A:G mutations. G:dT is ∼3 fold higher than C:dA in the Forward strain while C:dA is ∼3 fold higher in the Reverse strain. Comparing these data with the published *in vitro* results is also consistent with Polδ being responsible for replicating the lagging strand template. Strand dependence was not observed in *polδ^+^* (Table 3), indicating that the bias seen in *polδ -L591M* cells reflects base misincorporation by the mutant polymerase rather than sequence context or the transcriptional direction of marker genes. We did not observe strong hotspots for particular mutations, but the total number of occurrences is higher for some mutations, e.g., T to C at *ura4* base pair 236 and 76 in the Forward background and C to T at *ura4* 190 and (−91) in the Reverse background (Figure 3).

**Table 2:** Strand bias of mutants from *polδ-L591M.*

Mutation	Mispair*	in vitro**	Forward(lagging strand)	Reverse(leading strand)
AT>GC	A:dCT:dG	118∼44	225	104
GC>AT	G:dTC:dA	∼21	155	827
GC>TA	G:dAC:dT	7∼141	94	313
AT>TA	A:dAT:dT	2∼121	41	73
AT>CG	A:dGT:dC		04	40
GC>CG	G:dGC:dC		20	00
ΔAT	ΔAΔT	1∼5	923	1013
ΔGC	ΔGΔC	∼171	118	77

Strand bias seen in the *pol*δ*-L591M* mutant in the Forward and Reverse *ura4^+^:ura5^+^* backgrounds. Data from lines 1 and 2 are plotted in Figure 1F.

*Expected mispairs during synthesis of the transcribed strand.

**expected numbers based on in vitro analysis of Polδ L612M from Nick McElhinny SA *et al.* [6]

**Table 3:** Lack of strand bias of mutants from *polδ* ^+^.

Mutation	Mispair*	Forward(lagging strand)	Reverse(leading strand)
AT>GC	A:dCT:dG	10	35
GC>AT	G:dTC:dA	1310	2217
GC>TA	G:dAC:dT	2012	296
AT>TA	A:dAT:dT	43	23
AT>CG	A:dGT:dC	17	25
GC>CG	G:dGC:dC	01	04

Lack of significant strand bias of mutations observed from the *pol*δ^+^ strain in the Forward and Reverse *ura4^+^:ura5^+^* backgrounds

### Characterization of the *polε-M630F* Mutant

**Figure 4:**
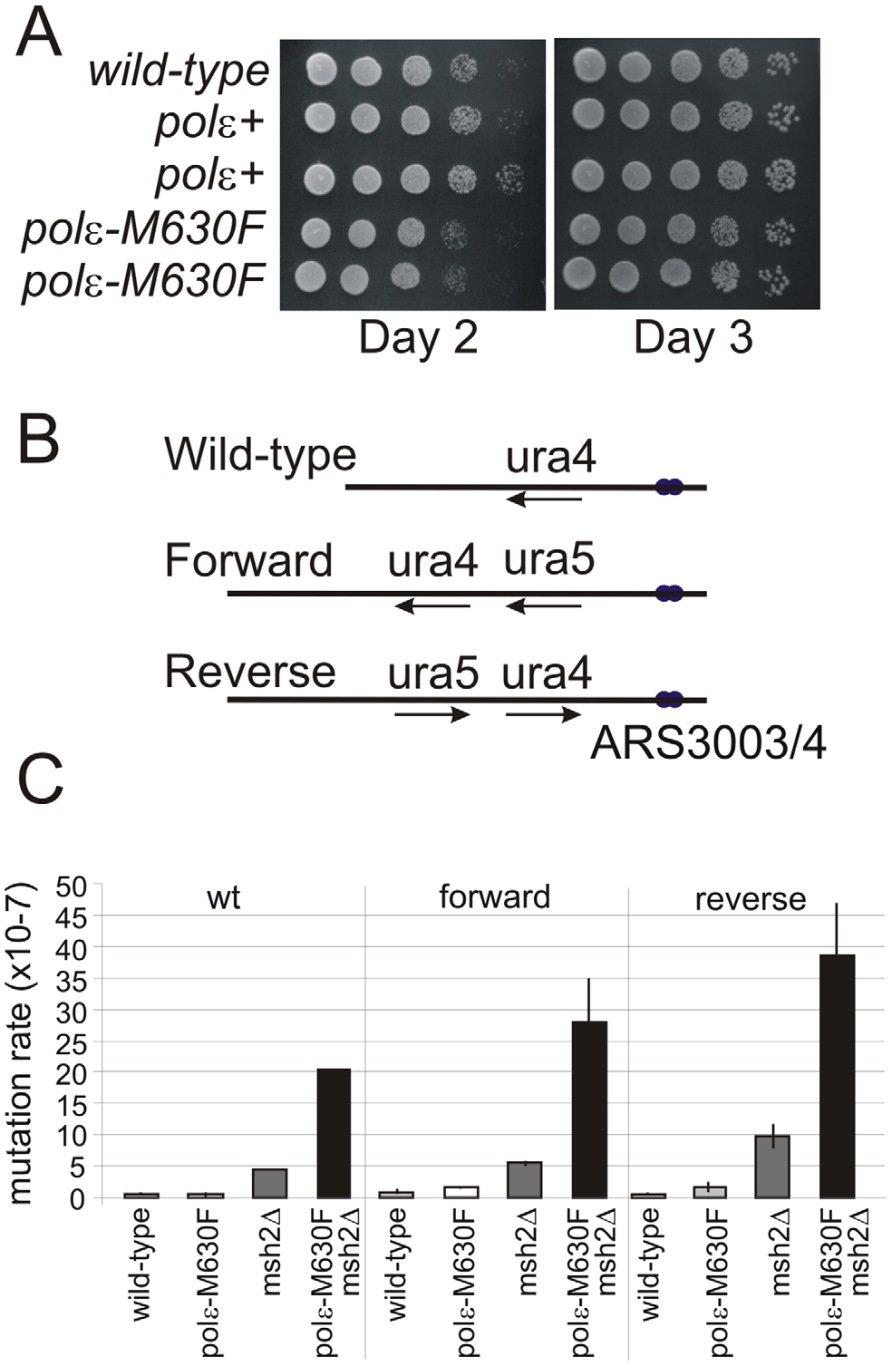
Mutation rate and dNMP incorporation by *polε-M630F*. A. Cell growth of wild type cells and *polε^+^* (wild-type *polε* flanked by lox sites) and *polε-M630F* strains. Serial dilutions of cells were spotted on YEA plates, incubated (30°C) for 2 or 3 days and photographed. B. Schematic of the wild type *ura4^+^* locus and the two versions of the modified *ura4^+^:ura5^+^* loci. C. Spontaneous mutation rates for *polε^+^*, *msh2*Δ, *polε-M630F* and *polε-M630F msh2*Δ double mutant cells in the *ura4^+^* (wild type), Forward and Reverse backgrounds. Error bars are standard deviations.

*S. cerevisiae* Polε M644G shows strong bias between A:dA and T:dT mispairs *in vitro* and the spontaneous mutation rates of the corresponding mutant cells are significantly higher than that of wild type and exhibit strand bias [8]. However, we found that the equivalent *Schizo. pombe polε-M630G* mutation is lethal, as was a *polε-M630K* mutation. Analysis of strains expressing Polε M630G or Polε-M630K from an ectopic integrated copy in a *pol*ε^+^ background (Figure S1) suggest this is largely due to catalytic inactivity, as mutation frequencies were not dramatically increased. Thus, we created strains harboring *polε-M630F* as an alternative. The decision to substitute to phenylalanine was based on earlier studies showing that Polα L868F is error prone in vitro and mutagenic *in vivo* [13], Polε M644F is error-prone *in vitro* with a weak bias in error rates [7], and Polζ L979F is error prone *in vitro* [24] and mutagenic *in vivo* [14]. The *Schizo. pombe polε-M630F* that we created using the RMCE methodology grows slightly more slowly than *polε^+^*, although the size of mutant colonies becomes comparable to that of wild type after prolonged incubation (Figure 4A). Strains harboring *polε-M630F* did not exhibit a substantial increase in spontaneous mutation rate in a mismatch repair proficient background. In strains wherein mismatch repair is inactivated by deleting the *msh2* gene, *polε-M630F* increased the mutation rate by 4–5 fold (Figure 4C). However, upon sequencing *ura5* and *ura4* from 5-FOA resistant clones, a strand bias sufficient to infer which strand is copied by the mutant Polε was not observed (Table S2).

### rNMP Incorporation into DNA by Polε M630F

**Figure 5:**
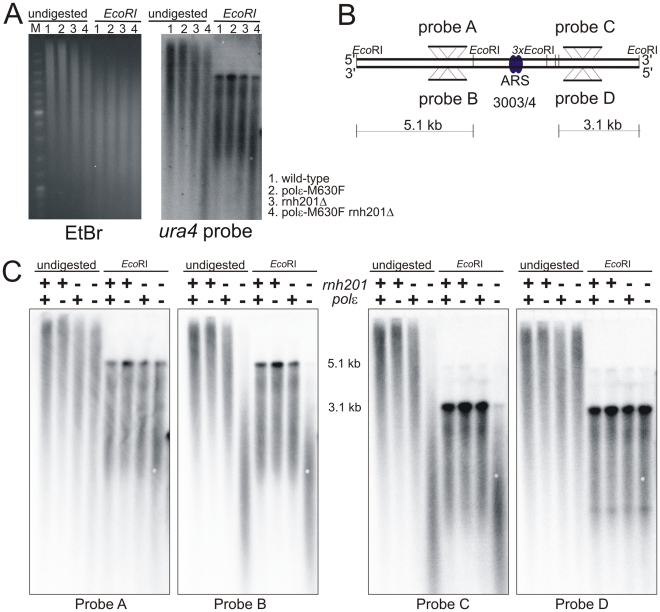
Strand bias for rNMP incorporation. A. Alkali sensitivity of genomic DNA. Genomic DNA from the indicated strains was either digested with *Eco*RI or left undigested, then treated with alkali and separated by alkaline agarose gel electrophoresis. DNA was revealed with ethidium bromide following neutralization and then processed for Southern analysis and probed with a *ura4* probe that reveals both DNA strands. B. Schematic of the loci either side of *ars3003/3004* indicating the positions of the *Eco*RI sites, plus the location and strand specificity of the probes used. C. Alkali sensitivity of each strand, either on the left of *ars3003/3004* (probes A and B) or on the right (probes C and D). Strains were either *polε^+^* (+) or *pol*ε*-M630F* (−) with or without concomitant deletion of *rnh201*, as indicated. The membrane from Figure 3D was stripped and hybridized with the indicated single stranded probes. Probe A and C hybridize with the top strand, probes B and D hybridize with the bottom strand.

Mutations at Polε M644 in *S. cerevisiae* affect the rate of rNMP incorporation into DNA [15]. We thus tested this possibility in *Schizo. pombe*. rNMPs incorporated into DNA are rapidly excised by the activity of RNase H2, whose catalytic subunit is encoded by the *rnh201* gene of *Schizo. pombe*. Since increased rNMP incorporation increases alkali-dependent DNA fragmentation, we assayed for increased gel mobility of DNA from the endogenous *ura4* ^+^ locus using Southern blot analysis. As anticipated, genomic DNA prepared from *polε-M630F* was not particularly sensitive to alkali treatment when compared to genomic DNA from the *polε^+^* strain (Figure 5A, lanes 1 and 2). However, it becomes significantly sensitive compared to *polε^+^* when *rnh201* is deleted (lanes 3 and 4). This indicates that Polε M630F incorporates rNMP into DNA at higher rate than wild type Polε and that these are largely removed by RNase H2 activity.

Based on this observation, we chose to test the strand specificity of rNMP incorporation using alkali treatment and subsequent probing for either the leading or lagging strand using the appropriate single-stranded probes. We prepared two pairs of probe across *ars3003/3004* (Figure 5B). The top strand is detected by probe A and C, while the bottom strand is detected by probe B and D. As shown in Figure 5C, only one of each of the two strands from *rnh201Δ polε-M630F* was sensitive to alkali at each probed site. The alkali sensitive strand was the bottom strand on the left side of the origin, while the top was sensitive on the right side (Figure 5B and 5C). Since those probed sites are inferred to be copied by replication forks emerged at *ars3003/3004*, the alkali-sensitive strands correspond to the nascent leading strand products of replication. Similar results were obtained at another origin (Figure S2). These results strongly suggest that Polε replicates the leading strand template.

## Discussion

An understanding of the fundamental mechanism of DNA replication is an important aspect of appreciating how replication and the errors made during replication influence evolution and human disease. While we have a breadth of knowledge about the proteins involved in eukaryotic DNA replication, including those that move with the active replisome, we do not have an unambiguous view of the architecture of the replication machine itself. Indeed, only recently has genetic data from the budding yeast *S. cerevisiae* linked the key replicative polymerases Polε and Polδ to leading and lagging strand synthesis, respectively. While these assignments are consistent with a number of additional observations, such as the role of Polδ in the maturation of lagging strands [25]–[27], it is important to provide additional evidence to reinforce these assignments, as well as to establish if they are evolutionarily conserved.

### Lagging Strand Synthesis by Polδ

To investigate the role of *Schizo. pombe* Polδ during DNA replication, we created strains that replicate using a Polδ L591M mutant protein. We showed that Polδ L591M is highly mutagenic and induced various types of mutations in *Schizo. pombe*. Strand dependence in transition mutations allowed us to conclude that the main role of Polδ is during lagging strand synthesis (Figure 2 and Table 2). However, the mutational bias seen in this mutant is weaker than would be predicted from the *in vivo* and *in vitro* studies of the equivalent *S. cerevisiae* mutant. Because we used mismatch repair proficient cells for this study (the double mutant was lethal), the mutation spectra we observed here reflect mispairs that have escaped mismatch detection and repair. This may influence our interpretations. For example, bacterial MutS protein has variable affinity for different mismatches, with G:T being one of the best substrates [28], [29]. Thus, the specificity of mismatch repair might have partially masked the bias of misincorporation induced by Polδ L591M. It is also possible that the mutation spectra were affected by spontaneous base damage that results in mismatches that escape mismatch repair. These caveats mean that, while our results are consistent with a function of Polδ as a lagging strand polymerase, we cannot exclude the possibility that Polδ partly participates in leading strand synthesis or that Polε (or indeed other polymerases) may partially replicate the lagging strand [10], [30].

In addition to point mutations that were expected from *in vitro* studies of *S. cerevisiae* and human polymerases, we also observed significantly enhanced formation of deletions and duplications in *polδ -L591M* cells (Table 1). All deletions and duplications occurred at repetitive DNA sequences. The majority of duplications involved <100 bases (Table S1), reminiscent of the mutation spectra for *S. cerevisiae rad27* mutants. Jin *et al.* showed that duplication rates were enhanced by mutations in the Polδ exonuclease domain [25] and *S. cerevisiae polδ -L612M* cells require functional Rad27 for viability [31]. These studies are consistent with our observations and add support to the premise that Polδ is involved directly in lagging strand synthesis in *Schizo. pombe*.

The size of deletions we observed was relatively larger than that of duplications. More than half of the deletions were loss of >100 bp of sequence. Cai *et al.* have observed that exonuclease deficient *E. coli* DNA polymerase II generates similar deletions flanked by direct repeat sequences [32]. They proposed a model in which a mismatch made by a mutator polymerase during replication of the first direct repeat promotes primer relocation to the second direct repeat. Furthermore, we observed a low frequency of inversions flanked by inverted repeat sequences and most of these inversions were associated with deletion, duplication, and/or gene conversion. These events can be explained by template switching. Taken together, these observations suggest that a mismatch formed during DNA replication can cause various kinds of genome rearrangements. Interestingly, chromosome abnormalities such as chromatid breaks are substantially elevated in *Pold1^+/L604G^* and *Pold1^+/L604K^* mouse cells [33].

### Leading Strand Synthesis by Polε

To investigate a role of Polε during normal DNA replication, we utilized the observation that *S. cerevisiae* Polε M644G increased rNMP incorporation [15], [34]. We first demonstrated that *Schizo. pombe polε-M630F* cells incorporate rNMP into DNA at higher frequency than *polε^+^* cells (Figure 5A). This property of the mutant polymerase made it possible to determine the strand that is copied by the mutant Polε. Incorporation of rNMP in the leading strand was strikingly higher in *polε-M630F* mutant cells compared to *polε^+^* cells (Figure 5B and Figure S1). This result strongly suggests that Polε synthesizes the leading strand. On the other hand, we failed to observe a significant difference in rNMP incorporation in the lagging strand. This suggests that Polε has, at most, a limited role in lagging strand synthesis.

*Schizo. pombe* cells that harbor *polε-M630G* were not viable, while the corresponding mutation does not cause lethality in *S. cerevisiae*. Interestingly, the N-terminal catalytic domain of Polε can be entirely deleted in both yeasts [35], [36], while a catalytically dead Polε, that retains the full-length protein, is inviable. Our mutation frequency analysis of cells expressing Polε-M630G in a *polε* ^+^ background (Figure S1) suggest the inviability of *polε-M630G* is because the corresponding protein is catalytically dead, rather than because it increases the mutation burden beyond that which is sustainable.

### Incorporation and Repair of rNMPs in DNA

In addition to supporting a role for Polε in leading strand replication, the results in Figure 5 extend to *Schizo. pombe* two important conclusions derived from earlier studies in *S. cerevisiae*, namely that large numbers of rNTPs can be incorporated into the nascent leading strand during replication without strongly affecting growth (Figure 4A) and the rNMPs that are stably incorporated into the *Schizo. pombe* genome by a eukaryotic replicase are efficiently repaired in a RNase H2-dependent manner. In *S. cerevisiae*, unrepaired rNMPs in DNA promote formation of short deletions between short, tandemly repeated DNA sequences, by a mechanism that is unaffected by mismatch repair status [34] and is initiated by topoisomerase 1-dependent cleavage of rNMPs [37]. Many of the deletions occur in a manner that depends on the orientation of the reporter gene in relation to the closest origin of replication [15], indicating that they result from rNMPs incorporated into the nascent leading strand by Polε. The characteristics of the *Schizo. pombe polε-M630F* strains described here offer the opportunity to determine if these consequences are conserved in fission yeast, and to also test whether mating type switching, which depends on rNMPs in DNA [38], is affected by increased rNMP incorporation by replicases and/or by RNase H2 or topoisomerase status.

### DNA Replication in *Schizo. pombe*

In this study, we examined roles of Polδ and Polε during normal DNA replication in *Schizo. pombe* using two different methods. The first method was a genetic analysis of mutation spectra asymmetry in *polδ* mutant cells. The second was a physical rNMP incorporation assay using *polε* mutant cells. The combination of these analyses indicates that genomic DNA is replicated in *Schizo. pombe* in similar manner as has been suggested for *S. cerevisiae*. Because *Schizo. pombe* and *S. cerevisiae* are highly diverged in evolutionary terms [39], [40] our results strengthen the interpretation that replication in all eukaryotes follows similar rules. We also add a physical assay to the previous genetic data, increasing the likelihood that the interpretation of the genetics is indeed correct. We mainly examined DNA replication at the genomic *ura4* locus, because replication initiation at this locus is known to be highly efficient (Figure 1B). However, a similar result was obtained for a second independent locus using the physical method for assigning Polε activity (Figure S1). Thus, it is reasonable to suggest that DNA replication occurs in similar manner throughout the genome. However, it remains possible that cells utilize these two polymerases in a different manner in some specific situations or at some specific loci.

## Materials and Methods

### *Schizo. pombe* Strains, Media, and Methods

*Schizo. pombe* cells were grown in yeast extract (YE) medium. Standard genetic and molecular procedures were employed as described previously [41]. To examine cell growth on plates, serial dilutions of cells were spotted on YEA (YE agar) plates, and incubated at 30°C.

### Generating DNA Polymerase Mutant Strains

The *cdc6^+^* and *cdc20^+^* genes were amplified by PCR and cloned into pUC19. *cdc6-L591F* and *cdc20-M630F* mutant genes were constructed by PCR-meditated site-directed mutagenesis and sequenced to ensure that only the desired mutation was introduced. Both wild-type and mutant genes were introduced into *Schizo. pombe* at their native loci by recombination-mediated cassette exchange (RMCE) [17].

### Determining Spontaneous Mutation Rates

Spontaneous mutation rates were determined by fluctuation assay as described previously [42]. Briefly, 11 independent single colonies were suspended in 5 ml YEP (YE+polypeptone) medium and grown to saturation at 30°C. Cells were diluted appropriately and plated on YEA or YEA containing 0.1% 5-fluoroorotic acid (5-FOA). Colonies were counted after 4 days incubation at 30°C. Mutation rates were calculated by the method of median [43]. Genomic DNA from a single 5-FOA resistant colony was isolated and the *ura5-ura4* construct was amplified by PCR to be sequenced.

### 2-D Gel Analysis

Directional 2-D gel analysis was performed as described previously [22] with modifications. Genomic DNA was extracted and digested with *Hind*III and *Blp*I as described in [44]. After the first dimension electrophoresis, DNA was digested with *Spe*I in a gel slice and subjected to the second dimension electrophoresis. Replication intermediates were detected by Southern blot.

### Detecting Alkali-Sensitive Sites in Genomic DNA

Genomic DNA was extracted from exponentially growing cells and purified by Qiagen genomic-tip 100/G. 5 µg of undigested or *Eco*RI digested DNA was incubated in 0.3 M NaOH at 55°C for 2 hours and subjected to 1% alkaline agarose gel electrophoresis [15]. Gels were neutralized and stained with ethidium bromide, followed by Southern blot.

### Southern Blotting

Southern blotting was performed according to [45]. DNA fragments of interest were amplified by PCR from *Schizo. pombe* genomic DNA and used as templates to obtain labeled probes. Radioactive nucleotides were incorporated into DNA using Ready-To-Go DNA Labeling beads (GE Healthcare) or strand specific primers and TaKaRa Ex Taq (TAKARA BIO).

## Supporting Information

Figure S1 Ectopic expression of polymerase epsilon mutants. A. Schematic of the loci where polε^+^ or mutant versions are expressed from the *cdc20* (Polε) promoter following integration downstream of *ura4* ^+^. B. Mutation frequencies of indicated strains, either with or without mismatch repair. C. 1. Protein levels of wild type GFP-tagged Polε expressed from the *cdc20* locus. 2–4, protein levels of GFP-tagged polε^+^ and indicated mutants expressed at the ectopic locus in a Polε^+^ background.
(TIF)

Figure S2 Strand Bias for rNMP Incorporation at *ade6* locus. A. Schematic of the loci either side of *ars3045* (Heicheinger et al, 2006. EMBO J. 25, 5171–5179) indicating the positions of the *Bam*H1 sites, plus the location and strand specificity of the probes used. C. Alkali sensitivity of each strand, either on the left of *ars3035/3036* (probes E and F) or on the right (probes G and H). Strains were either *polε^+^* (+) or *pol*ε*-M630F* (−) with or without concomitant deletion of *rnh201*, as indicated. Probe E and G hybridize with the top strand, probes F and H hybridize with the bottom strand.
(TIF)

Table S1 Size of deletion or duplication seen in the *polδ-L591M* mutant in the *ura4^+^:ura5^+^* backgrounds.
(DOC)

Table S2 Lack of significant strand bias of mutations observed from the polε-M630F strain in the Forward and Reverse *ura4^+^:ura5^+^* backgrounds. *Expected mispairs during synthesis of the transcribed strand. **expected numbers based on in vitro analysis of Polε M644F from Pursell ZF *et al.* [7].
(DOC)

## References

[1] Garg P, Burgers PM (2005). DNA polymerases that propagate the eukaryotic DNA replication fork.. Crit Rev Biochem Mol Biol.

[2] Johnson A, O'Donnell M (2005). Cellular DNA replicases: components and dynamics at the replication fork.. Annu Rev Biochem.

[3] Kunkel TA (2009). Evolving views of DNA replication (in)fidelity.. Cold Spring Harb Symp Quant Biol.

[4] Kunkel TA, Bebenek K (2000). DNA replication fidelity.. Annu Rev Biochem.

[5] Nick McElhinny SA, Gordenin DA, Stith CM, Burgers PM, Kunkel TA (2008). Division of labor at the eukaryotic replication fork.. Mol Cell.

[6] Nick McElhinny SA, Stith CM, Burgers PM, Kunkel TA (2007). Inefficient proofreading and biased error rates during inaccurate DNA synthesis by a mutant derivative of Saccharomyces cerevisiae DNA polymerase delta.. J Biol Chem.

[7] Pursell ZF, Isoz I, Lundstrom EB, Johansson E, Kunkel TA (2007). Regulation of B family DNA polymerase fidelity by a conserved active site residue: characterization of M644W, M644L and M644F mutants of yeast DNA polymerase epsilon.. Nucleic Acids Res.

[8] Pursell ZF, Isoz I, Lundstrom EB, Johansson E, Kunkel TA (2007). Yeast DNA polymerase epsilon participates in leading-strand DNA replication.. Science.

[9] Burgers PM (2009). Polymerase dynamics at the eukaryotic DNA replication fork.. J Biol Chem.

[10] Kunkel TA, Burgers PM (2008). Dividing the workload at a eukaryotic replication fork.. Trends Cell Biol.

[11] Schmitt MW, Venkatesan RN, Pillaire MJ, Hoffmann JS, Sidorova JM (2010). Active site mutations in mammalian DNA polymerase delta alter accuracy and replication fork progression.. J Biol Chem.

[12] Schmitt MW, Matsumoto Y, Loeb LA (2009). High fidelity and lesion bypass capability of human DNA polymerase delta.. Biochimie.

[13] Niimi A, Limsirichaikul S, Yoshida S, Iwai S, Masutani C (2004). Palm mutants in DNA polymerases alpha and eta alter DNA replication fidelity and translesion activity.. Mol Cell Biol.

[14] Sakamoto AN, Stone JE, Kissling GE, McCulloch SD, Pavlov YI (2007). Mutator alleles of yeast DNA polymerase zeta.. DNA Repair (Amst).

[15] Nick McElhinny SA, Kumar D, Clark AB, Watt DL, Watts BE (2010). Genome instability due to ribonucleotide incorporation into DNA.. Nat Chem Biol.

[16] Nick McElhinny SA, Watts BE, Kumar D, Watt DL, Lundstrom EB (2010). Abundant ribonucleotide incorporation into DNA by yeast replicative polymerases.. Proc Natl Acad Sci U S A.

[17] Watson AT, Garcia V, Bone N, Carr AM, Armstrong J (2008). Gene tagging and gene replacement using recombinase-mediated cassette exchange in Schizosaccharomyces pombe.. Gene.

[18] Fraser JL, Neill E, Davey S (2003). Fission yeast Uve1 and Apn2 function in distinct oxidative damage repair pathways in vivo.. DNA Repair (Amst).

[19] Dubey DD, Zhu J, Carlson DL, Sharma K, Huberman JA (1994). Three ARS elements contribute to the ura4 replication origin region in the fission yeast, Schizosaccharomyces pombe.. EMBO J.

[20] Patel PK, Arcangioli B, Baker SP, Bensimon A, Rhind N (2006). DNA replication origins fire stochastically in fission yeast.. Mol Biol Cell.

[21] Dai J, Chuang RY, Kelly TJ (2005). DNA replication origins in the Schizosaccharomyces pombe genome.. Proc Natl Acad Sci U S A.

[22] Friedman KL, Brewer BJ (1995). Analysis of replication intermediates by two-dimensional agarose gel electrophoresis.. Methods Enzymol.

[23] Venkatesan RN, Hsu JJ, Lawrence NA, Preston BD, Loeb LA (2006). Mutator phenotypes caused by substitution at a conserved motif A residue in eukaryotic DNA polymerase delta.. J Biol Chem.

[24] Stone JE, Kissling GE, Lujan SA, Rogozin IB, Stith CM (2009). Low-fidelity DNA synthesis by the L979F mutator derivative of Saccharomyces cerevisiae DNA polymerase zeta.. Nucleic Acids Res.

[25] Jin YH, Obert R, Burgers PM, Kunkel TA, Resnick MA (2001). The 3′→5′ exonuclease of DNA polymerase delta can substitute for the 5′ flap endonuclease Rad27/Fen1 in processing Okazaki fragments and preventing genome instability.. Proc Natl Acad Sci U S A.

[26] Garg P, Stith CM, Sabouri N, Johansson E, Burgers PM (2004). Idling by DNA polymerase delta maintains a ligatable nick during lagging-strand DNA replication.. Genes Dev.

[27] Pavlov YI, Frahm C, Nick McElhinny SA, Niimi A, Suzuki M (2006). Evidence that errors made by DNA polymerase alpha are corrected by DNA polymerase delta.. Curr Biol.

[28] Brown J, Brown T, Fox KR (2001). Affinity of mismatch-binding protein MutS for heteroduplexes containing different mismatches.. Biochem J.

[29] Su SS, Lahue RS, Au KG, Modrich P (1988). Mispair specificity of methyl-directed DNA mismatch correction in vitro.. J Biol Chem.

[30] Pavlov YI, Shcherbakova PV (2010). DNA polymerases at the eukaryotic fork-20 years later.. Mutat Res.

[31] Li L, Murphy KM, Kanevets U, Reha-Krantz LJ (2005). Sensitivity to phosphonoacetic acid: a new phenotype to probe DNA polymerase delta in Saccharomyces cerevisiae.. Genetics.

[32] Cai H, Yu H, McEntee K, Kunkel TA, Goodman MF (1995). Purification and properties of wild-type and exonuclease-deficient DNA polymerase II from Escherichia coli.. J Biol Chem.

[33] Venkatesan RN, Treuting PM, Fuller ED, Goldsby RE, Norwood TH (2007). Mutation at the polymerase active site of mouse DNA polymerase delta increases genomic instability and accelerates tumorigenesis.. Mol Cell Biol.

[34] Clark AB, Lujan SA, Kissling GE, Kunkel TA (2011). Mismatch repair-independent tandem repeat sequence instability resulting from ribonucleotide incorporation by DNA polymerase varepsilon.. DNA Repair (Amst).

[35] Kesti T, Flick K, Keranen S, Syvaoja JE, Wittenberg C (1999). DNA polymerase epsilon catalytic domains are dispensable for DNA replication, DNA repair, and cell viability.. Mol Cell.

[36] Feng W, D'Urso G (2001). Schizosaccharomyces pombe cells lacking the amino-terminal catalytic domains of DNA polymerase epsilon are viable but require the DNA damage checkpoint control.. Mol Cell Biol.

[37] Kim N, Huang SN, Williams JS, Li YC, Clark AB (2011). Mutagenic processing of ribonucleotides in DNA by yeast topoisomerase I.. Science.

[38] Vengrova S, Dalgaard JZ (2004). RNase-sensitive DNA modification(s) initiates Schizo. pombe mating-type switching.. Genes Dev.

[39] Kuramae EE, Robert V, Snel B, Boekhout T (2006). Conflicting phylogenetic position of Schizosaccharomyces pombe.. Genomics.

[40] Sipiczki M (2000). Where does fission yeast sit on the tree of life?. Genome Biol.

[41] Moreno S, Klar A, Nurse P (1991). Molecular genetic analysis of fission yeast Schizosaccharomyces pombe.. Methods Enzymol.

[42] Foster PL (2006). Methods for determining spontaneous mutation rates.. Methods Enzymol.

[43] Lea DaC CA (1949). The distribution of the numbers of mutants in bacterial populations.. J Genetics.

[44] Arcangioli B (1998). A site- and strand-specific DNA break confers asymmetric switching potential in fission yeast.. EMBO J.

[45] Lambert S, Watson A, Sheedy DM, Martin B, Carr AM (2005). Gross chromosomal rearrangements and elevated recombination at an inducible site-specific replication fork barrier.. Cell.

