# Supplementary figures and images for: The Major Roles of DNA Polymerases Epsilon and Delta at the Eukaryotic Replication Fork Are Evolutionarily Conserved
Source: PLoS Genet. 2011 Dec 1;7(12):e1002407. doi: 10.1371/journal.pgen.1002407 (PMC3228825; doi:10.1371/journal.pgen.1002407)

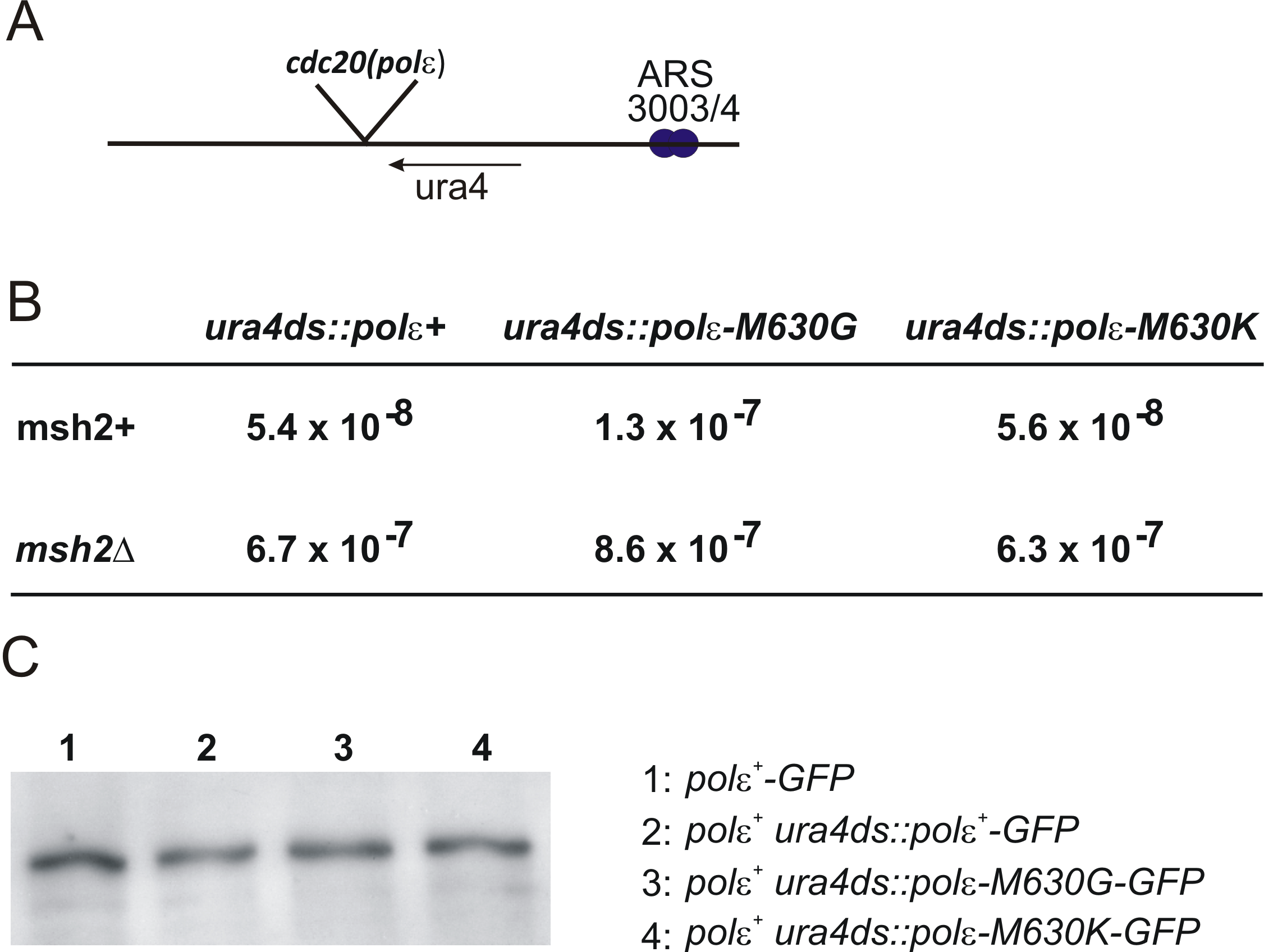

Supplement: Figure S1 — Ectopic expression of polymerase epsilon mutants. A. Schematic of the loci where polε+ or mutant versions are expressed from the cdc20 (Polε) promoter following integration downstream of ura4 +. B. Mutation frequencies of indicated strains, either with or without mismatch repair. C. 1. Protein levels of wild type GFP-tagged Polε expressed from the cdc20 locus. 2–4, protein levels of GFP-tagged polε+ and indicated mutants expressed at the ectopic locus in a Polε+ background. (TIF) [file pgen.1002407.s001.tif]

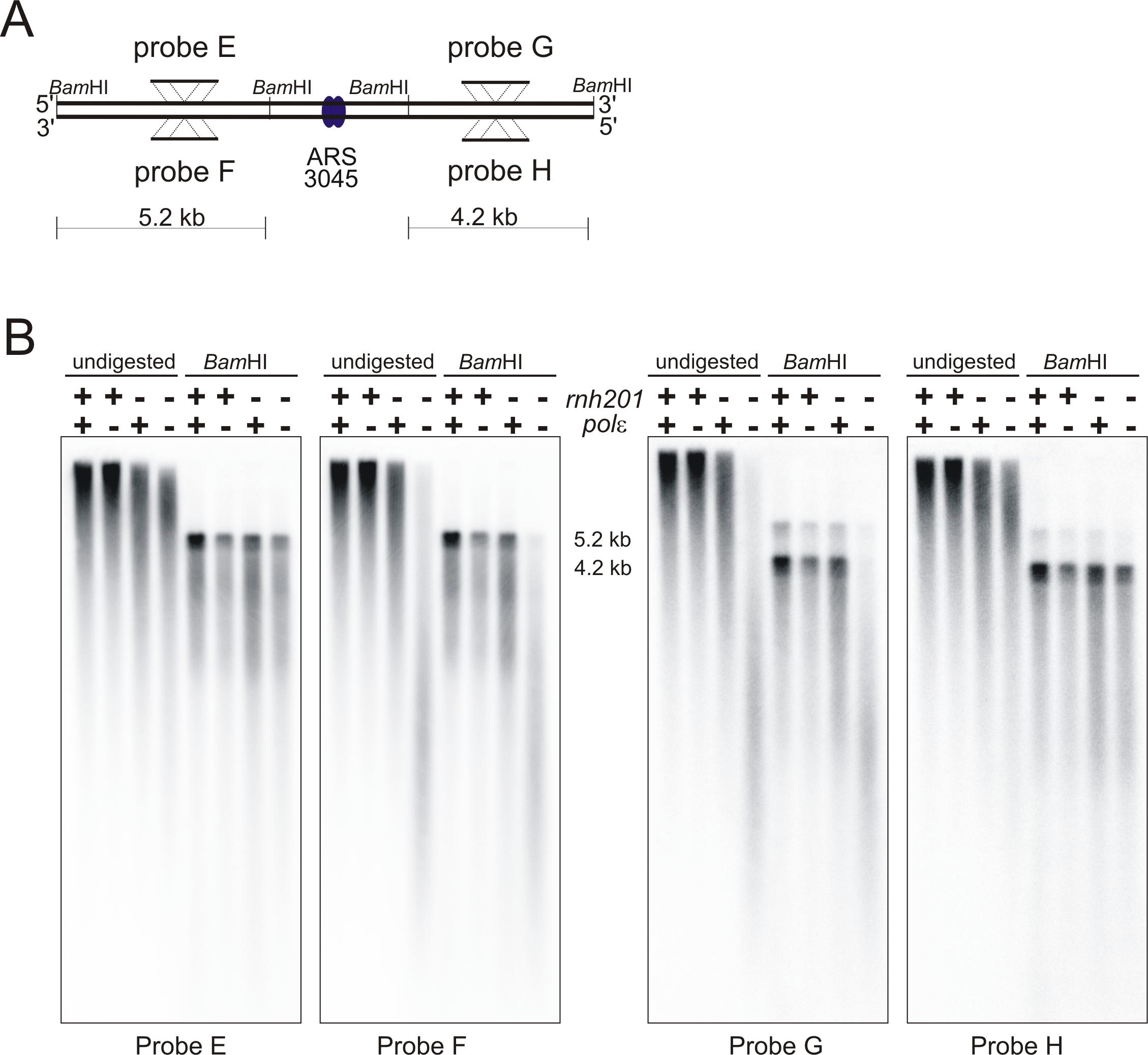

Supplement: Figure S2 — Strand Bias for rNMP Incorporation at ade6 locus. A. Schematic of the loci either side of ars3045 (Heicheinger et al, 2006. EMBO J. 25, 5171–5179) indicating the positions of the BamH1 sites, plus the location and strand specificity of the probes used. C. Alkali sensitivity of each strand, either on the left of ars3035/3036 (probes E and F) or on the right (probes G and H). Strains were either polε+ (+) or polε-M630F (−) with or without concomitant deletion of rnh201, as indicated. Probe E and G hybridize with the top strand, probes F and H hybridize with the bottom strand. (TIF) [file pgen.1002407.s002.tif]
