# Supplementary material for: The Major Roles of DNA Polymerases Epsilon and Delta at the Eukaryotic Replication Fork Are Evolutionarily Conserved
Source: PLoS Genet. 2011 Dec 1;7(12):e1002407. doi: 10.1371/journal.pgen.1002407 (PMC3228825; doi:10.1371/journal.pgen.1002407)
Supplement: Table S1 — Size of deletion or duplication seen in the polδ-L591M mutant in the ura4+:ura5+ backgrounds. (DOC) [file pgen.1002407.s003.doc]

Table S1

|  | Size of deletion/duplication (bp) | Occurrence |
| --- | --- | --- |
| Deletion | ≤100  >100 | 12 (41%)  17 (59%) |
| Duplication | ≤20  21-40  41-60  61-80  81-100  >100 | 5 (6%)  46 (52%)  9 (10%)  14 (16%)  9 (10%)  6 (7%) |
