# Supplementary material for: The Major Roles of DNA Polymerases Epsilon and Delta at the Eukaryotic Replication Fork Are Evolutionarily Conserved
Source: PLoS Genet. 2011 Dec 1;7(12):e1002407. doi: 10.1371/journal.pgen.1002407 (PMC3228825; doi:10.1371/journal.pgen.1002407)
Supplement: Table S2 — Lack of significant strand bias of mutations observed from the polε-M630F strain in the Forward and Reverse ura4+:ura5+ backgrounds. *Expected mispairs during synthesis of the transcribed strand. **expected numbers based on in vitro analysis of Polε M644F from Pursell ZF et al. [7]. (DOC) [file pgen.1002407.s004.doc]

Table S2

| Mutation | Mispair* | *in vitro*** | Forward  (lagging strand) | Reverse  (leading strand) |
| --- | --- | --- | --- | --- |
| AT->GC | A:dC | ~3 | 10 | 12 |
|  | T:dG | 1 | 11 | 9 |
| GC->AT | G:dT | 1 | 35 | 31 |
|  | C:dA | ~6 | 12 | 22 |
| GC->TA | G:dA | 1 | 44 | 12 |
|  | C:dT | ~1 | 3 | 21 |
| AT->TA | A:dA |  | 5 | 0 |
|  | T:dT |  | 1 | 1 |
| AT->CG | A:dG |  | 2 | 1 |
|  | T:dC |  | 0 | 3 |
| GC->CG | G:dG |  | 1 | 1 |
|  | C:dC |  | 4 | 9 |
| ∆A/T | ∆A |  | 24 | 38 |
|  | ∆T |  | 6 | 49 |
| ∆G/C | ∆G |  | 0 | 4 |
|  | ∆C |  | 11 | 59 |
